# Tear film characteristics in French bulldogs vs. non-brachycephalic dogs

**DOI:** 10.3389/fvets.2025.1732237

**Published:** 2025-12-10

**Authors:** Joschka Spornberger, Petr Soukup, Andrea Meyer-Lindenberg, Ingrid Allgoewer

**Affiliations:** 1 Animal Eye Practice, Berlin, Germany; 2 Clinic for Small Animal Surgery and Reproduction, Centre for Clinical Veterinary Medicine, LMU Munich, Munich, Germany

**Keywords:** brachycephalic dogs, evaporative dry eye disease, ocular surface health, tearfilm, tear film diagnostics

## Abstract

**Purpose:**

To investigate the tear film characteristics of healthy French Bulldogs and compare them with healthy dogs of mesocephalic and dolichocephalic breeds.

**Methods:**

French Bulldogs and non-brachycephalic dogs considered healthy underwent a complete ophthalmic examination. Tear osmolarity was tested with the ScoutPro® osmolarity system and a tear film analysis, including interferometry, non-invasive tear film break-up time (NIBUT), tear meniscus height measurement (TMH) and meibography using the I.C.P. OSA-Vet® was performed.

**Results:**

French Bulldogs had a significantly higher Schirmer Tear Test I (19.05 ± 4.00 vs. 16.88 ± 3.54, *p* = 0.017), a higher TMH (0.64 ± 0.20 vs. 0.31 ± 0.22, *p* = 9×10^−10^), a lower interferometry grading and a reduced NIBUT (7.39 ± 3.32 vs. 14.74 ± 5.92, *p* = 3.5×10^−9^) compared to the control group. Differences in tear osmolarity were not statistically significant between the groups (296.6 ± 13.6 vs. 299.8 ± 16.1, *p* = 0.40).

**Conclusion:**

The tear film in healthy French Bulldogs shows signs of a qualitative tear film deficiency/evaporative dry eye disease (EDED) compared to non-brachycephalic dogs. The influence of the altered tear film on diseases of the ocular surface in French Bulldogs needs to be further investigated.

## 1 Introduction

Brachycephalic dogs are disproportionately affected by ocular surface diseases (1–5). D. G. O’Neill et al. showed that brachycephalic dogs have a 3.63 times higher risk of developing keratoconjunctivitis sicca (KCS) than mesocephalic breeds (4). This, along with their overrepresentation in veterinary ophthalmology (6), is due to anatomic and physiologic changes that are collectively referred to as brachycephalic ocular syndrome (BOS) (1, 3, 7). However, there is discrepancy regarding the most common ocular surface disease in the various brachycephalic breeds (5–13) (Table 1).

**Table 1:** Breed specific prevalence of ocular surface disease in brachycephalic dogs: summary of published studies.

Breed	Number of animals/eyes	Health status	Most prevalent ocular surface disease	Study
Shih Tzu	1,012 eyes	ophthalmic disorders	KCS (12.5%)	Palmer et al. (6)
			Ulcer (11.1%)	
			Corneal pigmentation (10.1%)	
Pug	558 eyes	ophthalmic disorders	Corneal pigmentation (22.9%)	
			KCS (9.1%)	
Boston Terrier	567 eyes	ophthalmic disorders	Ulcer (14.8%)	
English Bulldog	320 eyes	ophthalmic disorders	Ulcer (13.4%)	
Lhasa Apso	201 eyes	ophthalmic disorders	KCS (16.9%)	
			Ulcer (11.9%)	
			Corneal pigmentation (7.5%)	
Pekingese	131 eyes	ophthalmic disorders	Corneal Pigmentation (17.6%)	
			Ulcer (17.6%)	
			KCS (15.3%)	
French Bulldog	84 eyes	ophthalmic disorders	Ulcer (20.2%)	
			KCS (4.8%)	
Pug	17 animals	ophthalmic disorders	Corneal pigmentation (53%)	Costa et al. (7)
Shih Tzu	22 animals	ophthalmic disorders	Corneal pigmentation (36%)	
Shih Tzu	22 animals	ophthalmic disorders	Fibrosis (36%)	
French Bulldog	38 animals	ophthalmic disorders	Fibrosis (29%)	
Shih Tzu	1,000 eyes	ophthalmic disorders	reduced tear film breakup time (17.6%)	Rajaei et al. (8)
			KCS (7.8%)	
			Corneal Pigmentation (7.8%)	
Shih Tzu	50 animals	healthy	Corneal Pigmentation (27%)	Sebbag et al. (9)
			Fibrosis (20%)	
			reduced tear film breakup time	
Shih Tzu	11 animals	KCS	Ulcer (36%)	Sanchez RF. et al. (13)
			Fibrosis (72%)	
Cavalier King Charles Spaniel	40 animals	KCS	Ulcer (30%)	
			Fibrosis (52.5%)	
Pug	295	ophthalmic disorders	Corneal pigmentation (82.4%)	
Pug	1,015 animals	ophthalmic and general disorders	Ulcer (5.42%)	O’Neill et al. (5)
Boxer	1,386 animals	ophthalmic and general disorders	Ulcer (4.98%)	
Shih Tzu	2031 animals	ophthalmic and general disorders	Ulcer (3.45%)	
Cavalier King Charles Spaniel	2,332 animals	ophthalmic and general disorders	Ulcer (2.49%)	
Bulldog	787 animals	ophthalmic and general disorders	Ulcer (2.41%)	
French Bulldog	642 animals	ophthalmic and general disorders	Ulcer (1.87%)	

Overview of studies on diseases of ocular surface diseases in brachycephalic dog breeds. For each breed, the number of animals or eyes examined, the general health status (e.g., presence of ocular diseases or healthy) and the most commonly reported ocular surface diseases are summarized. The data show breed-specific patterns in disease prevalence, particularly with regard to keratoconjunctivitis sicca (KCS), corneal ulcers, corneal pigmentation, and stromal fibrosis. The references refer to the original sources for each data set.

One of the brachycephalic breeds most commonly seen in specialist eye clinics is the French Bulldog. In 2024, 15.2% of all patients in a specialist eye clinic were French Bulldogs (14). Not only has their popularity increased in recent years, with registrations at the British Kennel Club rising by about 5.5 times between 2014 and 2021 (15), but they were the most popular dog breed in the United States in 2022 (16). In a retrospective study of 809 French Bulldogs, 76.6% showed keratitis (spontaneous chronic corneal epithelial defects (SCEED), 31%; melting ulcers, 19%; stromal keratitis, 16.3%; pigmentary keratitis, 5.3%; granulomatous keratitis, 5.2%) and 45% showed tear film disorders (quantitative KCS, 16.2%; qualitative KCS, 28.8%), underscoring the severity and frequency of ocular surface disease in this breed (17). Quantitative changes (aqueous deficient dry eye, ADDE) and qualitative changes in the tear film (evaporative dry eye disease, EDED) directly contribute to the development of ocular surface diseases (1). We hypothesized that French Bulldogs suffer disproportionately from qualitative tear film disorders.

The main objective of this study was to collect normative data on the characteristics of the ocular surface of French Bulldogs in order to advance research in the field of breed differences. The focus was on the examination of the tear film, which was evaluated using the I.C.P. OSA-Vet® and a series of additional tear film tests. These data were compared with those of non-brachycephalic breeds. Another objective was to investigate the usefulness of measuring osmolarity as an additional parameter for distinguishing between ADDE and EDED in veterinary medicine.

## 2 Materials and methods

The study population of this prospective study consisted of a group of healthy French Bulldogs and a control group of healthy non-brachycephalic breeds. French Bulldogs were included in the study if at least one eye was free of ocular disease and no topical eye medications had been administered within 5 days prior to the examination. The dogs were recruited either as inpatients with unilateral eye disease (with examination of the unaffected eye) or as clinically healthy pets from the same household. The control group consisted of mesocephalic and dolichocephalic dogs recruited from routine hereditary eye examinations, patients with unilateral eye disease, or healthy pets of the same household. In all cases, only eyes without clinical abnormalities and without recent topical treatment were included.

The owner’s consent for the evaluation of the anonymized data of their dog(s) and the photos taken for research purposes and publication was obtained prior to the examination. All examinations were carried out in accordance with the GERVO Declaration on the Use of Animals in Eye and Vision Research and with the approval of the Berlin State Secretariat for Health and Social Affairs under the number StN 024/22.

All dogs underwent a complete ophthalmic examination, including menace response, dazzle reflex, pupillary light reflex, palpebral reflex, slit lamp biomicroscopy (Kowa SL-17; Kowa, Japan), rebound tonometry (TonoVet, iCare, Finland), indirect ophthalmoscopy (Video Omega 2C; HEINE Optotechnik GmbH & Co. KG, Germany), a complete tear film analysis with the I.C.P. OSA-Vet® (SBM Sistemi, Italy), and a series of diagnostic tests for tear film deficiency, including osmolarity measurement, punctate fluorescein staining, lissamine green staining, Schirmer tear test I and Schirmer tear test II (all strips Madhu Instruments Pvt., Ltd., India). In 36 French Bulldogs and all non-brachycephalic dogs, tear film osmolarity was measured using the ScoutPro® osmolarity system (Trukera Medical, Southlake, USA). The tear film analysis with the I.C.P. OSA-Vet® was conducted first, followed by a 10 min rest period before the remaining examination.

### 2.1 Examination procedure

All examinations were performed by the same examiner (JS) in the same room. At the start of each examination, room temperature and humidity were recorded.

#### 2.1.1 Lipid layer thickness (LLT)/ interferometry (IF) grading

The interferometry measurement was performed using a short video clip of the eye surface after a blink, which was compared with the supplied video rating scale, taking into account the thickness of the lipid layer and thermodynamics (18). The rating scale comprised the following categories, as defined in I.C.P. OSA-Vet®: 0 = no lipid layer (lipid layer thickness < 15 nm), A = open meshwork (lipid layer thickness ≈ 15 nm), B = closed meshwork (lipid layer thickness ≈ 30 nm), C = wave pattern (lipid layer thickness ≈30–80 nm), D = amorphous pattern (lipid layer thickness ≈80 nm), E = color fringe (lipid layer thickness ≈80–120 nm) and F = abnormal color (lipid layer thickness ≈120–160 nm).

#### 2.1.2 Tear meniscus height (TMH)

The TMH was measured using the I.C.P. OSA-Vet® based on a photo taken after blinking to rule out inaccuracies due to evaporation of the tear film or trichiasis. The upper and lower limits of the tear meniscus were then marked manually on the photo and the TMH was calculated using the software provided. TMH was measured once at the central tear meniscus of the lower eyelid.

#### 2.1.3 Non-invasive breakup time (NIBUT)

The non-invasive tear film break-up time was measured using the I.C.P. OSA-Vet® based on projected Placido rings on the corneal surface. After manually blinking, the eye was kept open to record the video. The software measured the time until the rings deformed (18). In cases where automatic measurement was not possible (e.g., due to slight movements of the patient), the time was measured manually in the I.C.P. OSA-Vet® by marking the starting point after blinking and the end point when the first rings were deformed.

#### 2.1.4 Non-contact infrared meibography

The loss of Meibomian glands was measured using the I.C.P. OSA-Vet® by manually everting the tarsal plates of the upper and lower eyelids. Several images were taken and the area of the Meibomian glands was either marked by the software with manual control or marked manually. The software calculated the percentage loss of Meibomian glands (MGL) in relation to the tarsal plate (18).

#### 2.1.5 Osmolarity

The measurement was performed using the ScoutPro® osmolarity system on the tear meniscus of the lateral canthus in the lower eyelid area using disposable osmolarity test cards. The device collects 50 nanoliters of tear fluid and measures the osmolarity using a temperature-corrected impedance measurement (19). At the beginning of each day of testing, a quality control check was performed using the control card provided by the manufacturer.

#### 2.1.6 Schirmer tear test I (STT-I)

The Schirmer tear test I (Tear Touch Blu®, Madhu Instruments Pvt. Ltd., India) was performed by placing the strip in the lower lateral fornix of the eye. After one minute, the result was measured in mm/min.

#### 2.1.7 Schirmer tear test II (STT-II)

A drop of a topical anesthetic (proxymetacaine hydrochloride; Proparakain-POS 0.5%®, Ursapharm, Germany) was applied to the eye. After one minute, the excess liquid was carefully wiped off with a swab and the Schirmer tear test II was performed as described above for the Schirmer tear test I.

#### 2.1.8 Punctate fluorescein staining (PFS)

Fluorescein staining was performed by instilling a drop of a topical anesthetic (proxymetacaine hydrochloride; Proparakain-POS 0.5%®, Ursapharm, Germany) onto the eye via a fluorescein strip (Fluoro Touch®, Madhu Instruments Pvt., Ltd., India). The fluorescein was distributed by blinking several times manually. Excess fluorescein was washed out with sterile saline solution. The corneal surface was examined with a blue filter and 10x magnification using the Kowa SL 17 slit lamp. Classification was based on the evaluation system for surface area specified by Saito et al. (20) whereby grade 0 corresponds to no fluorescein uptake, grade 1 to uptake of less than 50% of the corneal surface, and grade 2 to uptake of more than 50% of the cornea.

#### 2.1.9 Lissamine green staining (LGS)

A lissamine green strip (Green Touch®, Madhu Instruments Pvt., Ltd., India) was moistened with a drop of topical anesthetic and applied once to the dorsal conjunctiva of the eyeball. The dye was distributed by manual blinking. The temporal conjunctival surface was examined using 10x magnification with the Kowa SL-17. Staining was graded as 0 = absent and 1 = positive. The severity of LGS is associated with reduced STT-I values (21) and the diagnostic options for evaporative dry eye are unclear (22). Since we did not expect low STT-I values and hypothezised qualitative tear film diosorders in the French Bulldog breed, no further differentiation was made.

### 2.2 Statistical analysis

The statistical analysis of the data was performed by Novustat GmbH (Wollerau, Switzerland). When both eyes of a dog were examined, the results were averaged (23). Bilateral differences were negligible and would not have been obscured by averaging. QQ plots indicate that the assumption of normally distributed data was not met. Therefore, the Wilcoxon test was used and *p* < 0.05 was considered statistically significant. Due to the presence of ties, the implementation of Hothorn et al. was used. In the case of ties, average ranks were calculated (24). Besides the uneven sample size, the loss of statistical power due to the unbalanced design is negligible (25). Since IF Grade and PFS are measured on an ordinal scale, Spearman’s rank correlation coefficient was used. The strength of the correlation was assessed using the following scheme: very weak (0–0.19), weak (0.2–0.39), moderate (0.40–0.59), strong (0.6–0.79), very strong (0.8–1.0) (26).

## 3 Results

### 3.1 Animals

Fifty client-owned French bulldogs (90 eyes) with an average age of 4.96 (0.5–12 years) and thirty client-owned non-brachycephalic breeds (58 eyes) were included in this study. Among the non-brachycephalic breeds were mixed breeds (n = 7), Shetland Sheepdog (n = 5), Golden Retriever (n = 4), Greyhound (n = 3), English Cocker Spaniel (n = 2), Polish Lowland Sheepdog (n = 2), Siberian Husky (n = 1), Saluki (n = 1), Greater Swiss Mountain Dog (n = 1), Whippet (n = 1), Flat-Coated Retriever (n = 1), Rough Collie (n = 1), Terrier (n = 1) and Australian Cattle Dog (n = 1). The average age of the non-brachycephalic dogs was 4.88 years (0.6–10 years). The French Bulldogs consisted of 17 females, 10 spayed females, 19 males, and 4 neutered males. The control group consisted of 13 females, 6 spayed females, 6 males, and 5 neutered males.

### 3.2 Ophthalmic examination

Based on the results of the ophthalmological examination, eight eyes (SCCED n = 2; ulceration n = 3, eyelid margin mass n = 1, dermoid n = 1, keratoconjunctivitis sicca n = 1) from the French Bulldog group and two eyes (reactive conjunctivitis n = 1, eyelid margin mass n = 1) from the control group were excluded from the study. Two French Bulldogs were presented after a previous unilateral enucleation.

### 3.3 Age

Age showed a weak negative correlation with STT-I (*r* = −0.32; *p* = 0.026) and osmolarity (*r* = −0.38; *p* = 0.021).

### 3.4 STT-I and STT-II

**Figure 1:**
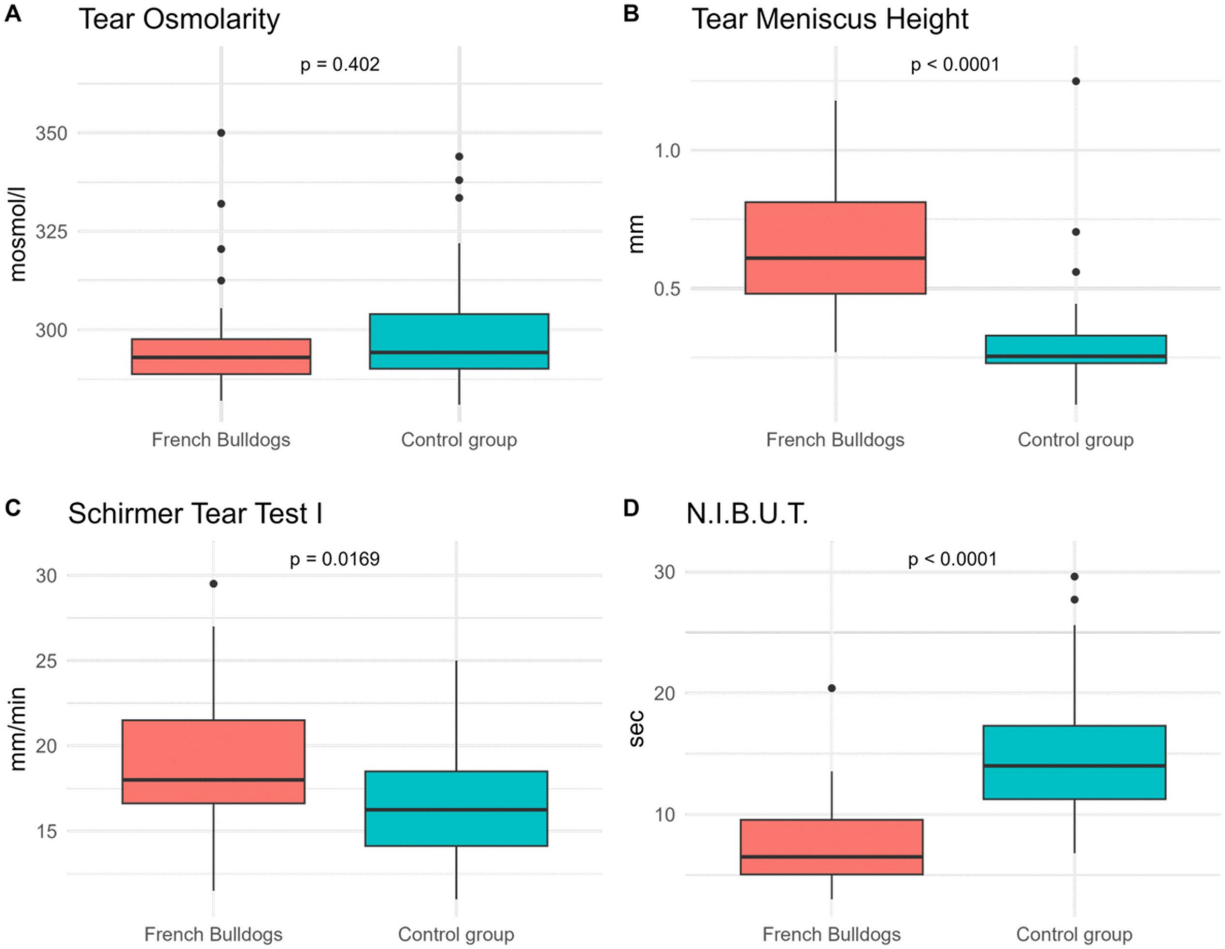
Box plots illustrating the distribution of tear film parameters in French Bulldogs (FB) and non-brachycephalic control dogs. The variables shown include: **(A)** tear osmolarity (mOsm/L); **(B)** tear meniscus height (mm); **(C)** Schirmer tear test I (mm/min); **(D)** non-invasive tear film breakup time (NIBUT; sec.). Each box represents the interquartile range between the 25th and 75th percentiles, with the horizontal line within the box indicating the median. The whiskers extend to the most extreme data points within 1.5 times the IQR, and individual points represent outliers outside this range.

The STT-I values were 19.05 ± 4.00 mm/min in French Bulldogs and 16.88 ± 3.54 mm/min in the control group. French Bulldogs had a significantly higher STT-I value (*p* = 0.01) than non-brachycephalic breeds. The STT-II values were 10.16 ± 2.86 mm/min in French Bulldogs and 11.28 ± 2.60 mm/min in the control group (Figure 1). The difference was not significant (*p* = 0.06). STT-I showed a weak negative correlation (*r* = −0.24) with PFS.

### 3.5 Lipid layer thickness (LLT)/ interferometry (IF) grading

**Figure 2:**
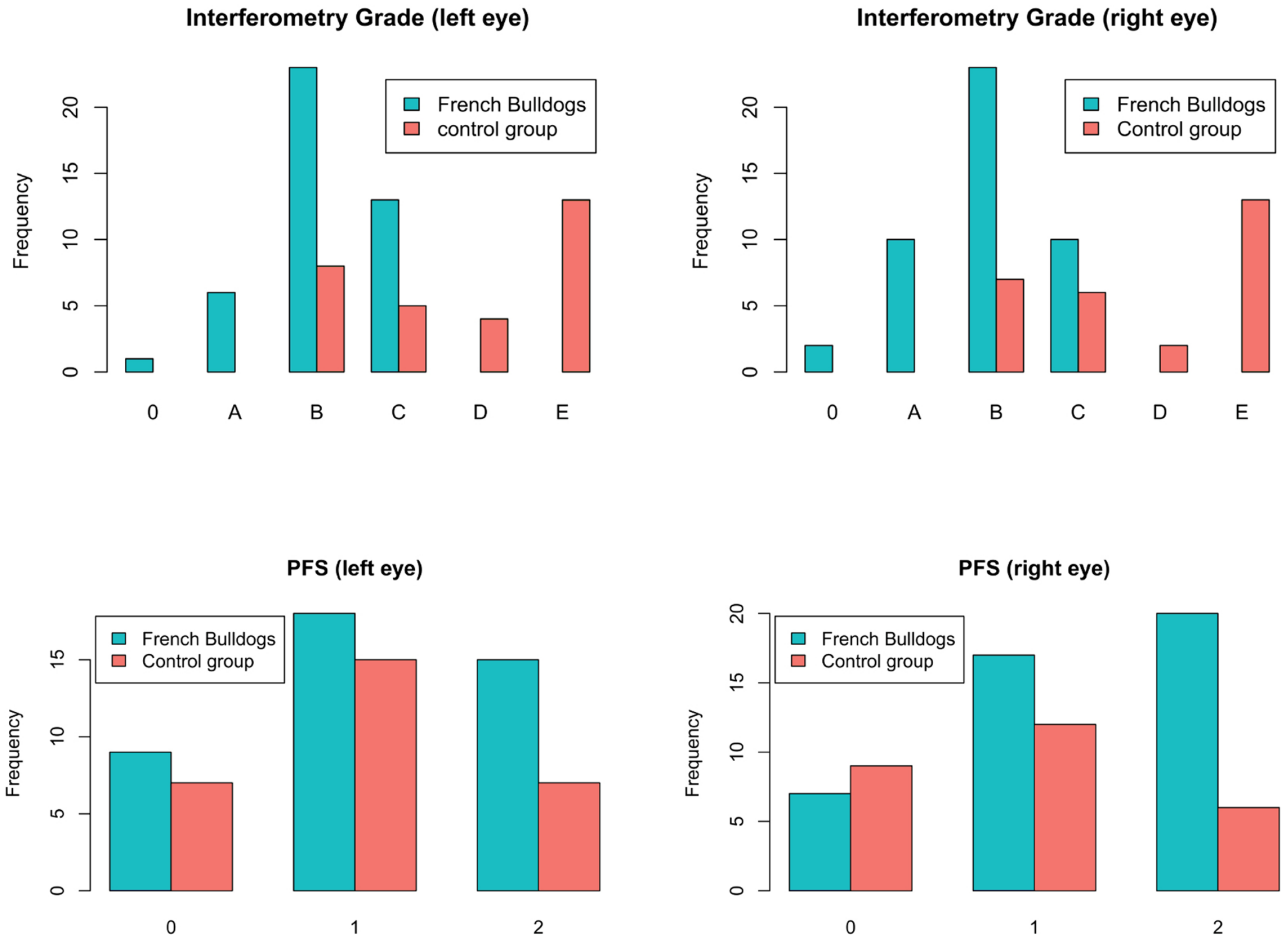
Distribution of interferometry grades (top row) and punctate fluorescein staining (PFS; bottom row) in the right and left eyes of French Bulldogs and non-brachycephalic control dogs. The interferometry grades represent lipid layer patterns from 0 (absent) to E (color fringe), based on OSA-Vet® classification; grade F (abnormal color pattern) is not displayed as it was not observed in any animal. PFS values are categorized as 0 (no staining), 1 (<50% of the corneal surface), and 2 (>50% of the corneal surface affected). Each parameter is reported separately for the left and right eye to account for possible unilateral deviations.

**Figure 3:**
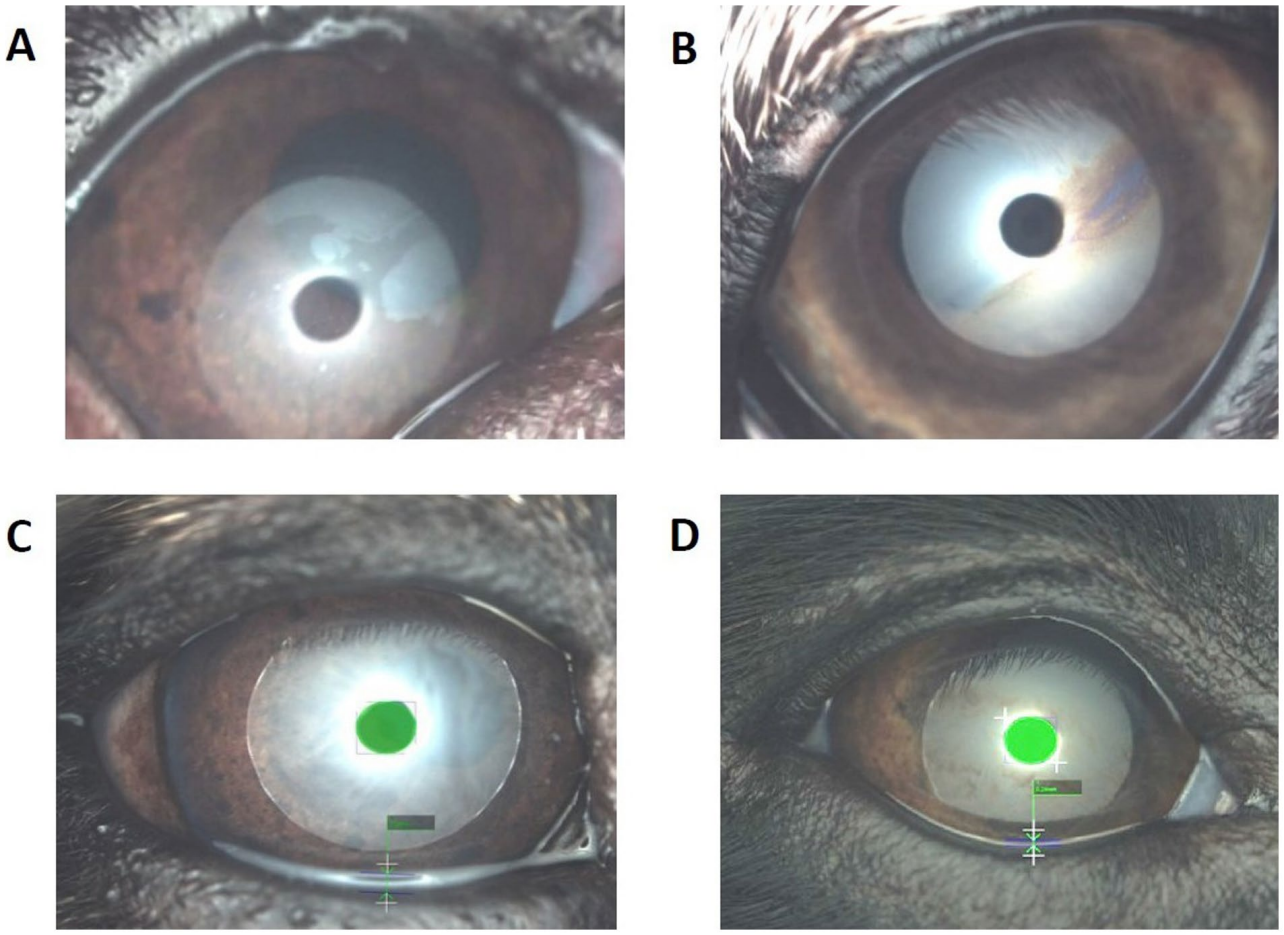
Representative I.C.P. OSA-Vet® images. **(A,B)** Interferometry of the precorneal tear film; **(C,D)** tear meniscus height (TMH) at the inferior central meniscus. **A** French Bulldog, 8 years old, open meshwork lipid-layer pattern (grade A). **B** Saluki, 7 years old, color fringe pattern (grade E). **C** French Bulldog, 9 years old, TMH 0.85 mm. **D** Greater Swiss Mountain Dog, 8 years old, TMH 0.26 mm.

No dog from either group achieved an IF score of F. French Bulldogs showed a significantly lower (*p* = 9*10^−8^) IF score than non-brachycephalic dogs. A comparison between the left and right eyes of both groups is shown in Figure 2. The IF grade showed a moderate positive correlation (r = 0.49) with NIBUT and a moderate negative correlation with the MGL of the upper eyelid (r = −0.42) and the lower eyelid (r = −0.46) (Figure 3).

### 3.6 Tear meniscus height (TMH)

The TMH values were 0.64 ± 0.20 mm in French Bulldogs and 0.31 ± 0.22 mm in the control group. French Bulldogs had a significantly (*p* = 9*10^−10^) higher TMH value than the control group (Figures 1, 3).

### 3.7 Non-invasive breakup time (NIBUT)

The mean NIBUT values were 7.39 ± 3.32 s in French Bulldogs and 14.74 ± 5.92 s in the non-brachycephalic group. French Bulldogs had a significantly (*p* = 3.5 × 10^−9^) lower NIBUT than the non-brachycephalic group. NIBUT showed a strong negative correlation with the MGL (r = −0.65) (Figure 1). In some French Bulldogs, mucous secretions or reduced patient compliance interfered with automatic NIBUT measurement, requiring manual assessment.

### 3.8 Non-contact infrared meibography

**Figure 4:**
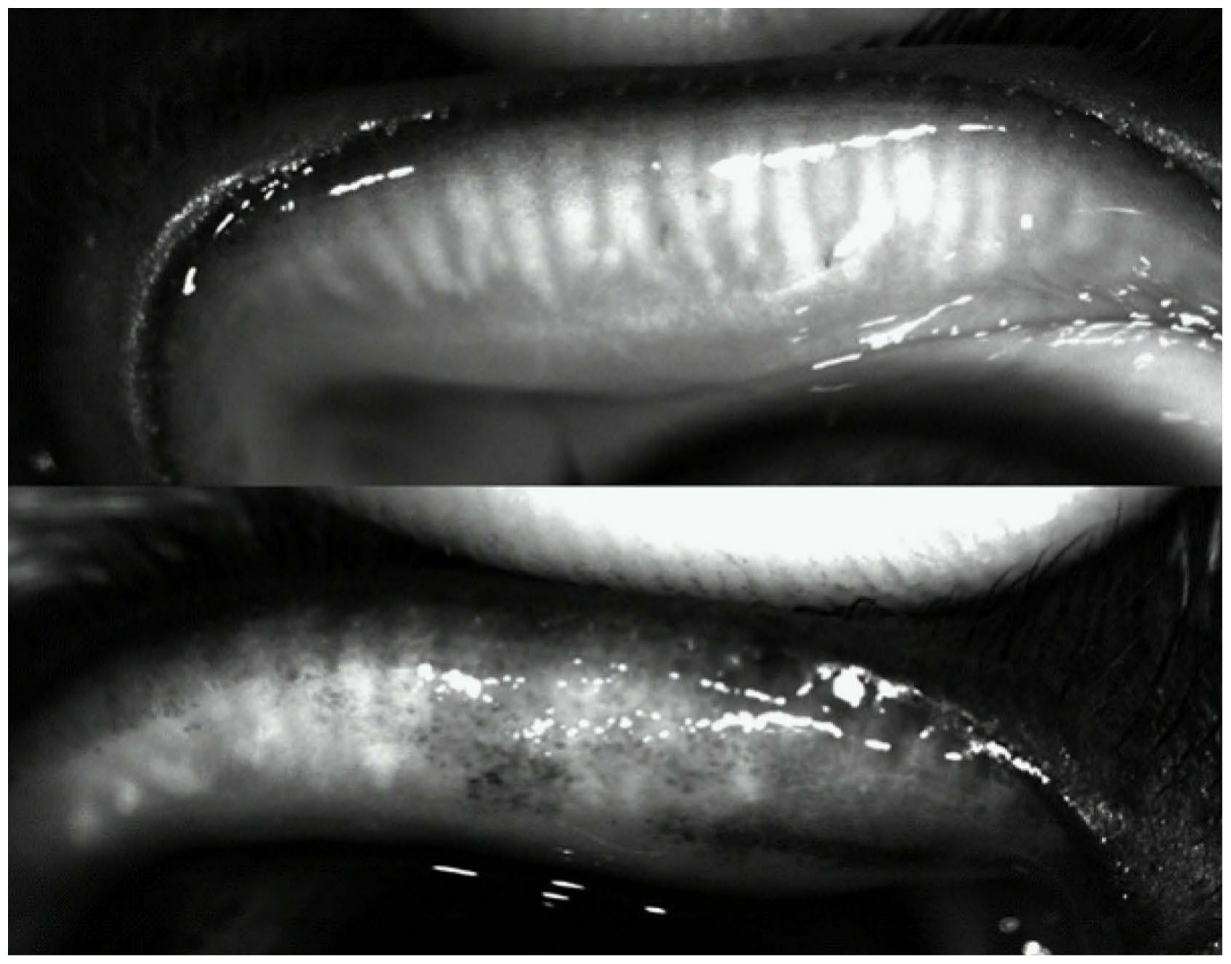
Infrared meibography images taken during examination with the I.C.P. OSA-Vet®. Top: Complete view of the Meibomian glands in the upper eyelid. Bottom: Partial obstruction of gland visualization due to pigmentation of the conjunctiva, as is often seen in French Bulldogs.

The Meibomian gland loss was measured for the upper eyelids. The mean MGL was 25.32 ± 19.11% in French Bulldogs and 14.57 ± 9.32% in the control group, showing a significant (*p* = 0.027) difference between the two groups. Minor difficulties were encountered with meibography. The presence of conjunctival pigmentation in some French Bulldogs (Figure 4) led to partial coverage of the Meibomian glands, which impaired image sharpness and subsequent analysis.

### 3.9 Osmolarity

The osmolarity was 296.6 ± 13.6 mOsm/L in the French Bulldogs and 299.8 ± 16.1 mOsm/L in the control group. The difference was not significant (*p* = 0.4) (Figure 1).

### 3.10 Punctate fluorescein staining (PFS)

The PFS showed no significant (*p* = 0.1) differences between the French Bulldogs and the control group (Figure 2). A weak negative correlation (*r* = −0.38; *p* = 0.0075) was found between the PFS and the IF score.

### 3.11 Lissamine green staining (LGS)

No LGS uptake was detected in the French bulldog group or in the control group.

A tabular overview of the most important parameters is provided in Table 2.

**Table 2:** Tear film parameters: French Bulldogs vs Controls.

Variable	French Bulldogs (*n* = 50)	Control group (*n* = 30)	*p*-Value between the groups
STT-I (mm/min)	19.05 ± 4.00	16.88 ± 3.54	0.01
STT-II (mm/min)	10.16 ± 2.86	11.28 ± 2.60	0.06
TMH (mm)	0.64 ± 0.20	0.31 ± 0.22	9×10^−10^
NIBUT (sec)	7.39 ± 3.32	14.74 ± 5.92	3.5×10^−9^
Osmolarity (mOsm/L)	296.6 ± 13.6*	299.8 ± 16.1	0.4
MGL upper eyelid (%)	25.32 ± 19.11	14.56 ± 9.32	0.027

Comparison of tear film parameters between French Bulldogs (*n* = 50) and non-brachycephalic control dogs (*n* = 30). Values are given as mean ± standard deviation. Statistically significant differences between groups were determined using the Wilcoxon test. *n = 36. STT-I, Schirmer tear test I; STT-II, Schirmer tear test II; TMH, tear meniscus height; NIBUT, non-invasive tear film breakup time; MGL, meibomian gland loss.

### 3.12 Room temperature and humidity

Room temperature for the French Bulldogs was 22.09 ± 1.93 °C and 21.54 ± 1.03 for the control group. There was no statistical difference between the two groups (*p* = 0.42). Humidity was 45.44 ± 8.79% for the French Bulldogs and 52.66 ± 5.56 for the control group. The difference was statistically significant with *p* = 0.0002. Room temperature and humidity showed a moderate positive correlation with NIBUT (*r* = 0.45; *p* = 0.001 and *r* = 0.54; *p* = 0.0001).

## 4 Discussion

To our knowledge, this is the first study to perform a comprehensive tear film analysis exclusively in French Bulldogs. While previous studies have examined certain brachycephalic breeds such as the Shih Tzu (8, 9) in great detail, the French Bulldog, despite being one of the most popular breeds in many countries (15, 16), has not been the subject of systematic tear film investigation to date. A number of studies (2, 4, 6, 7, 27) have included individual French Bulldogs in larger, heterogeneous cohorts. However, these data do not allow conclusions to be drawn about specific breeds. In contrast, the present study provides the first targeted insight into the tear film characteristics of this breed and highlights the clinical relevance of qualitative tear film abnormalities in otherwise ophthalmologically healthy dogs.

In this study, French Bulldogs showed a significantly higher STT-I value (*p* = 0.01) compared to non-brachycephalic breeds. The average STT-I values were 19.05 ± 4.00 mm/min in French Bulldogs and 16.88 ± 3.54 mm/min in the control group. This result contrasts with previous studies, which found STT-I values to be approximately 14% lower in brachycephalic dogs without clinical eye disease compared to non-brachycephalic dogs (1, 28). This discrepancy in these studies was primarily attributed to reduced corneal sensitivity and its negative effect on the afferent part of the tear reflex in brachycephalic breeds (1, 28). However, the results of this study suggest that this trend is not transferable to French Bulldogs. The increased STT-I in this breed is more likely related to increased reflex tear flow than to increased basal tear production. This assumption is supported by simultaneous evidence of a shortened NIBUT, reduced interferometry values, and a significantly increased TMH, all of which indicate EDED (1). It has been demonstrated that the process of increased tear evaporation leads to local dehydration of the cornea, activating both nociceptors and cold thermoreceptors in the cornea. This in turn stimulates the tear reflex (29, 30). The STT-I values observed in this study in French Bulldogs were similar to those reported by Li et al. for brachycephalic dogs (19.05 ± 4.00 mm/min vs. 18.2 ± 3.9 mm/min) (27), but lower than previously described for Shih Tzus (22.0 ± 5.5 mm/min) (9) and other brachycephalic dogs (20.1 ± 3.4 mm/min) (28). In contrast, STT-I values in the non-brachycephalic group showed a high correlation with data from the dolichocephalic subgroup (16.88 ± 3.54 mm/min vs. 16.8 ± 3.4 mm/min) from Li et al. (27).

A significant increase in TMH was observed in French Bulldogs compared to the control group (0.64 ± 0.20 mm vs. 0.31 ± 0.22 mm, *p* = 9 × 10^−10^), resulting in more than a twofold increase in TMH compared to control values. These results confirm the report by Li et al. on higher TMH in brachycephalic breeds (27). An increase in TMH could be a result of an impairment in the tear drainage system (31). However, although the tear drainage system of brachycephalic dogs is anatomically different, they consistently have an additional drainage into the oral cavity (32), thus supporting a normal tear drainage. The suggested normal range for TMH, measured with the I.C.P. OSA-Vet® in healthy dogs with normal STT-I, normal eyelid shape, and normal tear drainage system, is 0.53 ± 0.11 mm (18). This value is higher than the data reported by Li et al. of 0.27 ± 0.12 mm for mesocephalic breeds and 0.26 ± 0.09 mm for dolichocephalic breeds (27), 0.41 ± 0.21 mm by Kim et al. for Beagles, 0.32 ± 0.14 mm (33) and 0.36 ± 0.18 mm by Sonego et al. in their groups of non-brachycephalic breeds (34), the median values measured by Yoon et al. using optical coherence tomography of the anterior segment of the eye (0.32 mm and <0.32 mm) (35) and 0.31 ± 0.22 mm in this study. A study by Silva et al. found a TMH of 0.59 ± 0.29 mm in the right eye and 0.88 ± 0.27 mm in the left eye of healthy Shih Tzus (36). In a study on qualitative and quantitative tear tests in various brachycephalic breeds, Voitena et al. reported a TMH of 0.51 ± 0.17 (37). Zwiauer-Wolfbeisser et al. reported a TMH of 0.5 ± 0.2 mm, with the study population consisting of 58.3% brachycephalic breeds treated for distichiasis, making the population incomparable to the other studies (38). Peruccio et al. did not disclose the number of dogs and breeds examined (18). The available data on TMH shows that TMH is increased in brachycephalic breeds compared to non-brachycephalic breeds (27, 33–38). Our results suggest that the significant increase in TMH observed in French Bulldogs, combined with the significant decrease in NIBUT and lower interferometry values, provides convincing evidence of underlying EDED in this breed. A substantial variation in reported TMH is observed among studies involving a brachycephalic cohort. This variations includes lower (27, 37) and higher (36) values compared to the values reported in our study. This heterogeneity is likely attributable to variations in cohort composition. These findings underscore the importance of establishing breed-specific reference values for tear film diagnostics, particularly in brachycephalic dogs. The NIBUT was significantly lower in French Bulldogs than in the control group (7.39 ± 3.32 s. vs. 14.74 ± 5.92 s., *p* = 3.5*10^−9^). This result is consistent with other studies comparing brachycephalic breeds with non-brachycephalic breeds (27, 39). The shorter NIBUT could contribute to the higher STT-I, as reported by Faghihi et al. (39). The value for the control group was at the lower end of the reported normal range for tear film breakup time (TBUT) in healthy dogs (19.7 ± 5 to 21.53 ± 7.42 s.) (40). It was lower than the NIBUT in healthy beagles (33). Li et al. (27) and Voitena et al. (37) reported lower than previously described NIBUT values as well. Li et al. discussed that their lower NIBUT values may be due to environmental influences that contribute to a shorter break-up time, especially in the summer months (27). As reported in the results section, relative humidity was higher in the control group, whereas room temperature did not differ between groups. Because higher humidity reduces evaporative loss (41), this would be expected to prolong NIBUT in the control group and thereby exaggerate the observed between group difference. Although temperature can influence the tear film, temperature-related bias is unlikely in in this study due to comparable temperatures. Since all ambient values were within the typical indoor range (42), the influence is likely to be minor and probably not responsible for the general pattern of significantly shorter NIBUT in French Bulldogs. Nevertheless, residual bias cannot be completely ruled out.

The IF grade was significantly lower in French Bulldogs (*p* = 9*10^−8^) than in the control group, with the majority of French Bulldogs achieving an IF grade of B and the control group achieving a grade of E. The IF grade allows a direct estimation of the lipid layer thickness. The lipid layer is the outermost layer of the tear film, which prevents evaporation of the aqueous phase and stabilizes the tear film (43). It is produced by the Meibomian glands (43). Increased loss of the Meibomian glands or Meibomian gland dysfunction (MGD) leads to a reduction in the lipid layer (22) and thus to a low IF grade. This explains the correlation between a higher MGL and a lower NIBUT. A possible change in the composition of meibum due to MGD may also increase evaporative stress and provoke reflex tearing, which contributes to the higher STT-I and TMH, respectively (44). Another factor that must be taken into account is allergies, which are common in brachycephalic breeds (45). Studies in human medicine have shown that seasonal allergies alter the morphology of the Meibomian glands, promoting a change in lipid secretion and instability of the tear film (46, 47). In accordance with the previously mentioned findings, preliminary data reported by Marques et al. (ESVO 2025) revealed that atopic dogs suffering from allergic conjunctivitis exhibited lower IF grades in comparison with healthy controls. This observation supports to the hypothesis that ocular allergies are related to impairment of the lipid layer.

We have documented a higher MGL value in French Bulldogs, which could partly explain the lower IF grade. However, some patients had both a low IF grade and a low MGL percentage. Consequently, the MGL percentage is not the only factor influencing the IF grade. MGD is a multifactorial disease in which not only the end stage of glandular loss must be considered, but also qualitative and quantitative changes in the secreted lipid content. This may be due to hyposecretory, obstructive, or hypersecretory mechanisms (48, 49). As Ofri et al. have shown, there are differences in the amount of meibum secreted in different dog breeds (50). This also explains the prevalence of low IF grades in French Bulldogs. Another possible cause is incomplete closure of the eyelids during blinking, and the lower blink rate often observed in brachycephalic breeds (22). The reduced nerve fiber density of the cornea in brachycephalic dogs (51) is thought to be the cause of the reduced blink rate in these breeds (1). This leads to meibum not being distributed evenly across the cornea, which promotes destabilization of the tear film. Possible MGD in the French Bulldogs requires further investigation.

There are conflicting statements in the human literature as to whether only the upper eyelid (52), the lower eyelid (53), or both eyelids (54, 55) need to be measured for the correct diagnosis of Meibomian gland disease. Also, in the veterinary literature there is no consensus on which eyelid should be examined using meibography. Kim et al. performed meibography on both eyelids of Beagles and found it difficult to adequately expose the Meibomian gland area of the lower eyelid (33). Kitamura et al. did not examine the lower eyelid because this was found to be uncomfortable for their cohort of Shih Tzus (56). Li et al. examined the lower eyelids only because the patients showed discomfort during the examination of the upper eyelid (27). In our study, we selected the upper eyelid for meibography during the ophthalmological examination, as most dogs were more comfortable with it.

In this study, tear osmolarity was measured using the ScoutPro®. Veterinary clinical validation data for this device are limited (57). The osmolarity values of both groups were lower than previously reported normal values of TearLab® and I-PEN® Vet (58) and the ScoutPro® (57). Although reduced NIBUT and LLT values are typically associated with increased evaporation and increased osmolarity (29), the lower osmolarity of the French Bulldogs is presumably a consequence of the significantly increased STT-I and TMH values. This interpretation is supported by García-Resúa et al., who demonstrated a negative correlation between TMH and osmolarity (59). Given the lower osmolarity values in the control group, it can be concluded that a tear reflex triggered by handling can be ruled out, as no significant difference was found between the osmolarity of basal tears and reflex tears (60).

The clinical interpretability of osmolarity values remains unclear due to a lack of more published data for the ScoutPro® in dogs. After considering the above factors, it was determined that osmolarity within our specific cohort did not exhibit sensitivity to evaporation-related instability. Our observation shows that there was no difference in osmolarity between French Bulldogs and the control group. Consequently, osmolarity is considered an inadequate single marker for qualitative tear film deficiency in dogs. Further validation of the device and standardization of protocols are deemed necessary.

There were no differences between French Bulldogs and the control group regarding PFS and lissamine green staining. This is to be expected as STT-I was not decreased in animals included in this study. High PFS scores correlate with a lower STT-I (20). The weak negative correlation between PFS and IF grade aligns with the general findings, as PFS is regarded as a parameter for ocular health (22). Smith et al. reported higher lissamine green staining scores in dogs with lower STT-I values and shorter TBUT (21).

When interpreting the results, the limitations of the study must be taken into account. The assessment of the Meibomian glands was limited to non-contact infrared meibography. Image quality was partially limited by anatomical conditions or pigmentation. Although allergies are an important factor in MGD in humans, allergy testing was not performed in French Bulldogs, as this was beyond the scope of this study. All examinations and image analyses were performed by a single examiner without formal assessments of intra- or interobserver variability, which may limit reproducibility and introduce potential observer bias.

## 5 Conclusion

This study provides strong evidence that all clinically normal French Bulldogs in this cohort showed consistent indicators of EDED. Despite the absence of obvious clinical symptoms, the dogs showed a combination of shortened NIBUT, reduced LLT, increased TMH, and increased STT-I - characteristic features of a qualitative tear film disease. These findings suggest that EDED is not an isolated occurrence but rather a widespread condition within this breed. It is essential that routine ophthalmic examinations of French Bulldogs consider this underlying pathology.

## Data Availability

The original contributions presented in the study are included in the article/supplementary material, further inquiries can be directed to the corresponding author.
